# Eosinophilic Esophagitis: real-life outcomes over 10 years in a Canadian pediatric centre

**DOI:** 10.3389/fped.2025.1687724

**Published:** 2025-12-17

**Authors:** Nikola Deretic, Jonathan W. Bush, Stephanie C. Erdle, Edmond S. Chan, Vishal Avinashi

**Affiliations:** 1 Division of Gastroenterology, Hepatology and Nutrition, BC Children’s Hospital, University of British Columbia, Vancouver, BC, Canada; 2 Department of Pathology and Laboratory Medicine, BC Children’s Hospital, University of British Columbia, Vancouver, BC, Canada; 3 Division of Allergy, BC Children’s Hospital, University of British Columbia, Vancouver, BC, Canada

**Keywords:** Eosinophilic Esophagitis, pediatric, adherence, remission, real-life, Cow's Milk Protein Allergy, cohort, South Asian

## Abstract

**Objective:**

Despite Eosinophilic Esophagitis (EoE) being a chronic condition, many studies focus on the short-term. This study characterizes patients, treatment effectiveness and outcomes in a pre-biologic era.

**Methods:**

This cohort study (2012–2022) at British Columbia (BC) Children's Hospital in Vancouver, Canada analyzed data from the EoE Registry which was hosted on Research Electronic Data Capture (REDCap) for participating patients <18 years with biopsy-proven diagnosis (≥15 eosinophils/hpf) including demographics, symptoms, allergic history, medications, endoscopy and histology.

**Results:**

247 patients (71.2% White, 16.7% South Asian, 78.1% male, median age 9 years) were followed over a median 3-year follow up. 85.2% had at least one atopic condition and 17.4% reported Cow's Milk Protein Allergy in infancy. 19.0% lacked follow up endoscopy and in clinic follow-up, 18.2% were on no therapy. At last endoscopy of those on treatment (*n* = 200), 39.0% used swallowed topical corticosteroids (50.0% remission), 23.0% proton pump inhibitors (29.0% remission), and 39.0% elimination diets (34.0% remission). Over half on medications had imperfect adherence. Overall, 39.0% achieved remission (<15 eosinophils/hpf), (mean peak eosinophils decreased from 55 to 27/hpf, strictures from 7.5% to 2.7%).

**Conclusions:**

This Canadian study reveals remission rates lower than short term studies, but a reduction in strictures in follow-up. Real-life challenges, such as not returning for follow up endoscopy (∼20%), despite no direct care costs, challenges with adherence with ∼20% not taking any therapy and over half not taking medications as prescribed contribute towards remission rates below 50% regardless of treatment. Better patient engagement, addressing barriers to treatment and follow up and exploring novel therapies are needed.

## Introduction

Eosinophilic Esophagitis (EoE) is a chronic esophageal condition characterized by inflammation and caused by an antigen-mediated eosinophil response. EoE has only been well followed since the 1990s, with pediatric incidence and investigation of EoE increasing (1). The shortcoming of many EoE studies has been treatment efficacy in controlled settings for short time periods, but not effectiveness. Additionally, there have been few studies that have gathered real-life data on a pediatric cohort, with most studies analyzing charts retrospectively.

This unique study followed a pediatric cohort of 247 EoE patients over a decade and is amongst the largest reported cohorts in Canada. Our clinic has been running as a multidisciplinary clinic since 2012 and has involved gastroenterology, allergy, and dietitians. We were interested in describing our patient population over time, reflecting real life practice.

We analyzed data from our EoE Registry which collected data from patients who visited the British Columbia (BC) Children's Hospital EoE clinic over 10 years until 2022. We aimed to characterize patients who visited the clinic and describe selected treatments, and their effectiveness in a pre-biologic era. Additionally, we aimed to describe outcomes based on treatments at last follow-up, including macroscopic features such as strictures and histological activity as represented through peak eosinophil counts.

## Methods

This pediatric cohort study took place at BC Children's Hospital in Vancouver, Canada which is the only pediatric tertiary care centre in British Columbia. Inclusion criteria were any patient who was: age <18 years, a BC resident, experiencing upper GI symptoms preceding a biopsy-proven EoE diagnosis (≥1 biopsy showing ≥15 eosinophils/high powered field), and consenting to participate in the EoE Registry. Inflammatory Bowel Disease (IBD) patients were excluded from the study as IBD is a distinct entity and is on the differential for esophageal eosinophilia. Data for the EoE Registry was collected over 10 years (2012–2022) and included clinical, endoscopic, and histologic data about pediatric EoE patients seen in the EoE clinic. Data from 247 EoE patients was prospectively entered into the Registry, deidentified and securely stored on Research Electronic Data Capture (REDCap), a secure web-based data management software (2).

Basic demographic variables such as age, sex, and ethnicity were documented during the first EoE clinic visit. On intake, the child's symptoms that led to the EoE diagnosis, relevant past medical history, and immediate family history were captured by family (self) reported intake sheets. Participants left some questions blank on the intake sheets leading to a variable number of subjects answering certain questions. Allergy visits involved assessing food allergies and other atopic conditions. Concurrent Gastroenterology visits involved assessing current symptoms, medications, and dietary restrictions. Endoscopy results were analyzed at both initial and last follow-up endoscopy. Endoscopy and histology results included an assessment of macroscopic findings as well as microscopic features and eosinophil counts (at the proximal, mid, and distal esophagus). Treatments described include medications or diets patients were on at any point during the follow-up period. Remission was defined as having a peak eosinophil count <15 eos/hpf, which is the most common criteria amongst observational studies, while deep remission was defined as having a peak eosinophil count ≤6 eos/hpf, to capture more stringent histologic control (3). Macroscopic features captured include trachealization, exudate, furrowing and stricture.

Categorical variables are expressed as proportions. Quantitative variables are expressed as a median with an associated interquartile range (IQR). The analysis was descriptive (percentages, medians and means) and summarized cohort characteristics, treatments and outcomes given the real life heterogeneity of therapies and adherence.

## Results

Over the 10 years of data collection (2012–2022), 247 pediatric patients were enrolled in the study. Demographic data is summarized in Table 1. There was an average of 20.1 newly referred EoE patients per year. The median age at first visit was 9 (4.0, 13.0) years old and most patients were white (71.2%) and male (78.1%). The patient cohort was highly atopic, with 85.2% having had at least one atopic condition and 77.9% had an atopic condition within the past year. Before presentation to the EoE clinic, patients had a median of one visit (0.0, 2.0) to the emergency department for symptoms related to EoE. After presenting at the EoE clinic, patients had a median follow up of 3 years (IQR 1.1, 5.8) with a median of 6 total visits (3.0, 10.0) and 3 endoscopies (2.0, 5.0). 19.0% of patients only had one diagnostic endoscopy and no follow-up endoscopies.

**Table 1:** Demographic characteristics of children with EoE.

Characteristic	Number (%)
Age at first visit (median, IQR) (*n* = 247)	9.0 (4.0, 13.0)
Male sex (*n* = 247)	193 (78.1%)
Ethnicity	
White (*n* = 198)	141 (71.2%)
South Asian (*n* = 198)	33 (16.7%)
East Asian (*n* = 198)	2 (1.0%)
Other (*n* = 198)	22 (11.1%)
Atopic Disease (ever)	
At least one atopic condition (*n* = 236)	201 (85.2%)
Allergic rhinoconjunctivitis (*n* = 242)	143 (59.1%)
Atopic dermatitis (*n* = 242)	125 (51.7%)
Asthma (*n* = 241)	112 (46.5%)
Pollen-food allergy syndrome (*n* = 242)	49 (20.2%)
Food allergy (*n* = 236)	115 (48.7%)

**Figure 1:**
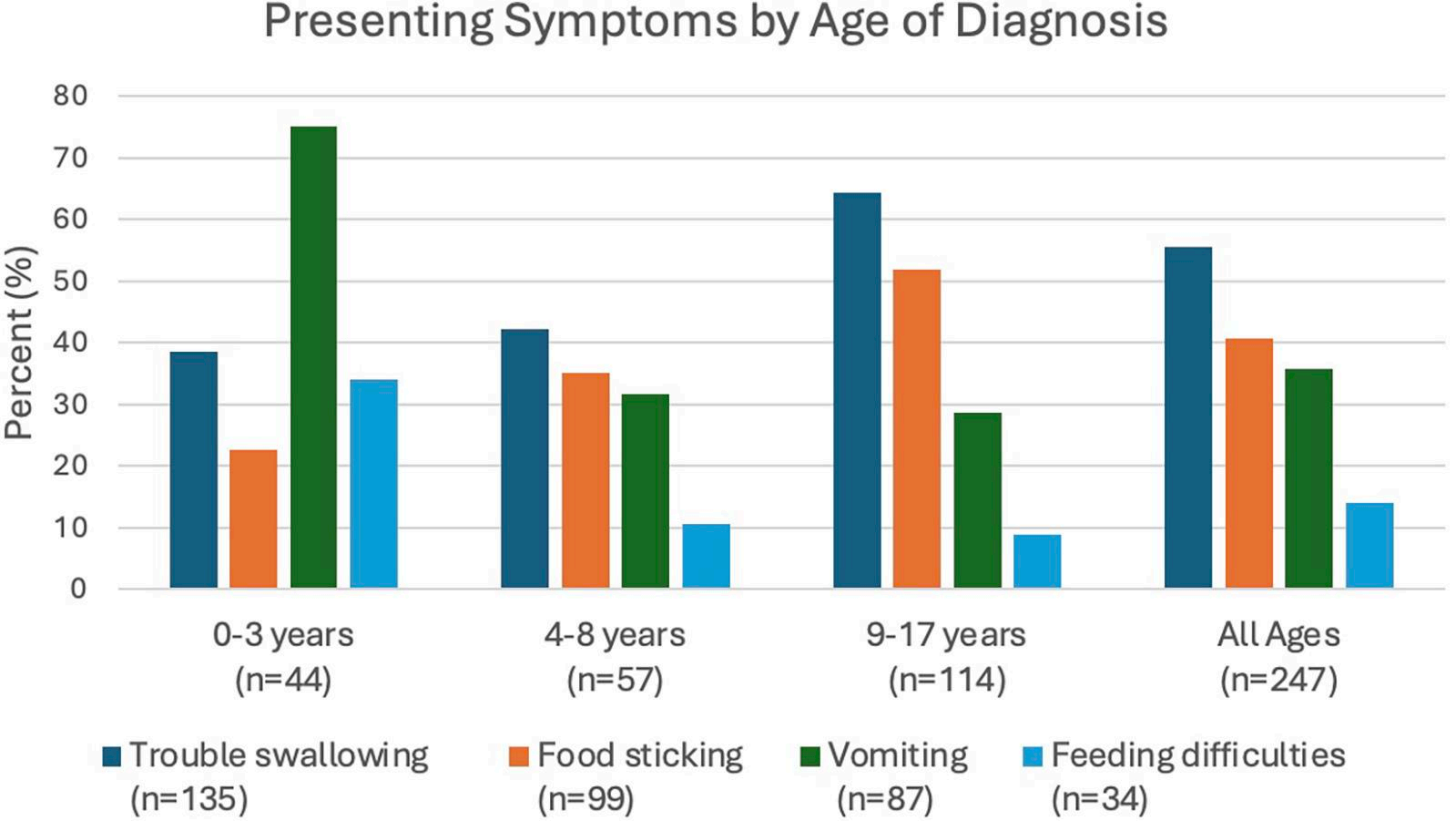
Presenting symptoms by age of diagnosis in children with EoE.

The most common symptoms for presenting at the clinic were trouble swallowing, food sticking, vomiting, and feeding difficulties (Figure 1). For those 0–3 years old, 75% presented with vomiting, which was higher than older children who had vomiting around 29%. Feeding difficulties were reported in 34.1% of children aged 0–3 years. Trouble swallowing was the most common presenting symptom for those 4–8 years old (42.1%) and 9–17 years old (64.3%). With regards to early life risk factors (Table 2), 39.3% of patients were born by c-section. In early life, 9.0% of patients were solely formula-fed, 35.9% received both formula and breast milk, and 55.2% were exclusively breastfed. In infancy, 15.3% of patients required a specialized formula while 17.4% were reported to have Cow's Milk Protein Allergy in infancy. Additionally, 23.9% of patients received antibiotics in the first two years of life, and approximately 32.4% had a history of using anti-reflux medication prior to diagnosis. Reported IgE-mediated food allergy was common in 48.7%, and 25.9% reporting immediate family history of food allergy. A total of 12.7% of patients reported a family history of first-degree members experiencing difficulty swallowing, vomiting, or food impaction, while 6.6% had a family history of diagnosed EoE.

**Table 2:** Early life risk factors including family history in children with EoE.

Risk factor/history	Number (%)
Past Medical History	
Early Life Indicators	
Birth by c-section (*n* = 150)	59 (39.3%)
Exclusive breastfeeding during first 6 months of life (*n* = 145)	80 (55.2%)
Used specialized formula as an infant (*n* = 137)	21 (15.3%)
Cow's Milk Protein Allergy reported as an infant (*n* = 236)	41 (17.4%)
Antibiotics in the first two years of life (*n* = 247)	59 (23.9%)
Medical History (Self-Reported)	
Acid reflux prior to EoE diagnosis (*n* = 236)	69 (29.2%)
Anti-reflux medication prior to EoE diagnosis (*n* = 247)	80 (32.4%)
Celiac disease (*n* = 236)	16 (6.8%)
Environmental allergies (*n* = 236)	48 (20.3%)
Family History (Self-Reported)	
Food allergy (*n* = 228)	59 (25.9%)
Celiac disease (*n* = 228)	15 (6.6%)
Acid reflux (*n* = 228)	42 (18.4%)
Trouble swallowing, vomiting and/or food getting stuck (*n* = 228)	29 (12.7%)
Eosinophilic Esophagitis (*n* = 228)	15 (6.6%)
Environmental allergies (*n* = 228)	51 (22.4%)

**Figure 2:**
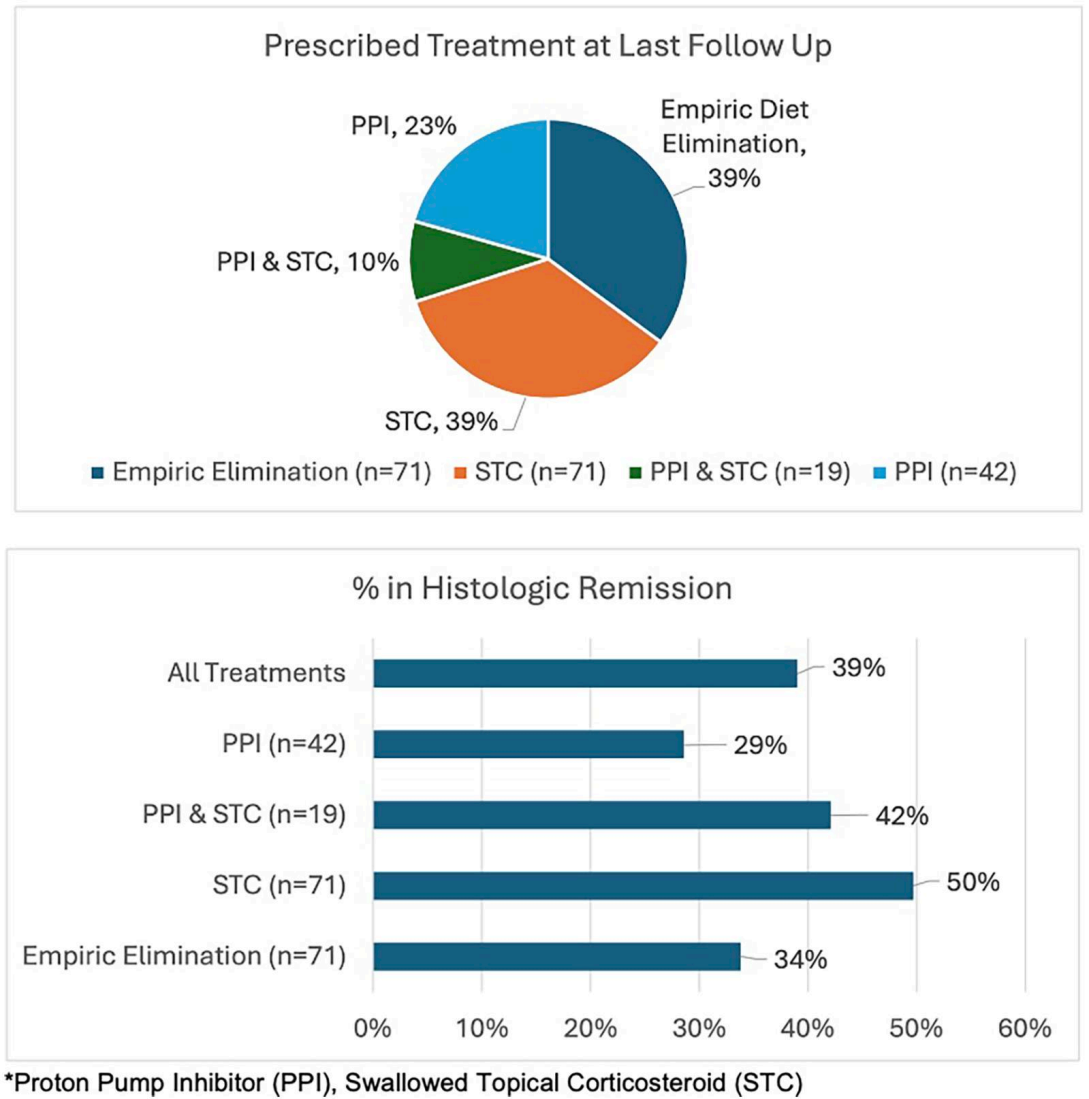
Prescribed treatment and response rate (<15 eos/hpf) at last follow Up in children with EoE.

The most common medications tried were PPI, swallowed topical corticosteroid (STC) [Fluticasone/Oral Viscous Budesonide (OVB)/budesonide orodispersible tablets] or a combination of both. 85.0% of patients tried at least one medication. Over half (51.9%) of patients tried at least one-food elimination diet and 14.2% tried more than a one-food elimination diet. By their last endoscopy (*n* = 200), (as seen Figure 2), 23.0% of patients were on PPI, 39.0% of patients were on STC and 10.0% were on a combination of the two. There were 39.0% that remained on an empiric elimination diet. By the last endoscopy, of the 71 patients on dietary elimination, 63.0% followed a cow's milk elimination diet, 3.0% a 4-food elimination diet, 6.0% a 6-food elimination diet, and 28.0% were on other combinations like two- or single-food eliminations such as wheat. The majority of patients (60.7%) were on no diet by the last endoscopy. There were 26.1% of patients that were not on any medication by the last endoscopy. During follow-up visits, 18.2% were on no treatment.

At the time of the last endoscopy, remission rates were variable (see Figure 2). Of those on a food elimination diet, 66.0% had active disease. Among the patients who were on a PPI during their most recent endoscopy, 71.0% exhibited active disease. In contrast, 50.0% of patients that were on a STC had active disease. Adherence was an issue with PPI being taken less often than prescribed on 55.5% of visits and not at all on 2.3% of visits. STCs were taken less often than prescribed for 54.7% of visits and not at all for 4.2% of visits. The most common reason for taking a PPI under half the time was “forgetfulness”, and “perceived side effects” was the biggest barrier towards STC adherence. Those in remission (maximum eosinophils <15 eos/hpf) tried a median of 2 medications compared to those with active disease that tried a median of 1 medication. In the most recent endoscopy involving 200 patients, 61.0% of the patients had active disease, while 39.0% were in remission (maximum eosinophils <15 eos/hpf), including 31.2% in deep remission (maximum eosinophils ≤6 eos/hpf). There was a decrease in peak eosinophil count from diagnosis by a median value of 29.5 eos/hpf between the initial and last endoscopy going from 55 to 27 eos/hpf. In terms of macroscopic features, from initial to last, furrowing decreased by 19.3% (78.1% to 58.8%), trachealization decreased by 6.0% (20.4% to 14.4%), and exudate decreased by 18.6% by the last endoscopy (44.3% to 25.7%). Strictures were defined for this study by the inability to pass a standard scope (∼9 mm) during endoscopic procedure. The rate of stricture decreased 4.8%, from 7.5% at initial endoscopy to only 2.7% by last endoscopy.

## Discussion

This study is important and unique as there are few studies tracking a pediatric cohort of this size over several years. Our single site Canadian study followed 247 patients over 10 years, where 200 patients had at least one follow up endoscopy (median of 3).While the majority of our cohort was white, similar to the existing literature (4), we had a higher proportion of South Asian (nearly 17%). Although there are more East Asian children than South Asian children in BC, there were very few East Asian children in our EoE cohort which is more likely to be due to some unique interplay between genetics and the environment rather than underdiagnosis of East Asian children with EoE (5). Our cohort was highly atopic with >85% having at least one atopic condition which is slightly more than in other pediatric study groups (80%, 78%) (6, 7).

In our cohort, 39.3% of patients were born via C-section, which is similar to the published provincial average of 37% (8). This is contrasting some literature identifying C-section as a risk factor for the development of EoE (9). Breastfeeding was not an obvious risk factor as our cohort reported the same (or slightly higher) rates of exclusive breastfeeding at 6 months (55.2%) vs. the provincial rate (48.2%) (10). Antibiotic exposure rates amongst our EoE patients of around 25% at 2 years is also comparable to provincial cohorts (11). About 1 in 6 in our cohort were reported to be using a specialized formula as an infant for CMPA. The general population has a lower rate of CMPA estimated at approximately 2%–3%, with even fewer requiring specialized formula (12). Although intriguing, these numbers are based on self-report with few patients having verified diagnoses, hence recall bias may be a factor.

EoE has been found to have a strong genetic component (13). In our study 6.6% of patients had a first degree relative reported to have EoE but about twice as many endorsed a first degree relative with symptoms suggestive of EoE. This lends further evidence that family members of pediatric EoE patients might have undiagnosed EoE.

Past reviews have explored the complex relationship between GERD and EoE (14). Prior to the AGREE consensus (15), patients were commonly prescribed PPIs before the diagnosis of EoE which may have screened PPI responders out and lowered response rates. By the last endoscopy, the most likely medication for patients to be on was a Swallowed Topical Corticosteroid (STC) and with our data it was demonstrated to be the most effective (around 50% remission). All treatments were less effective than previously published original articles (16–18).

Adherence is a significant real-life variable which affects efficacy with at least one half of our patients for both PPI and STC describing that their medication adherence is suboptimal. In clinic follow up, 1 in 5 patients despite being advised to be on some therapy, were on no therapy at all. Further, approximately 20% did not come back for repeat endoscopy despite no direct financial cost (physician visits and procedures are covered under our public health care system). Medication and diet nonadherence are both around 30% in the literature (19) but in our cohort, no comment can be made on cost barriers to treatment, as this was not explored.

Another more directed aspect of care which may impact real life effectiveness is our approach to target the lowest effective dose. A patient may respond to a higher induction dose but we often rescope on a lower maintenance dose with variable effectiveness. This is similar to other published dose reduction protocols (20).

With regards to dietary therapy, a recent review found that remission (<15 eos/hpf) in children demonstrated response rates over 50% regardless of the empiric elimination diet (21). Our rates were lower, however, our patients were most often doing a single food elimination diet (milk elimination) striking a balance of effort and feasibility, with a focus on sustainability (22). The principle of lowest effective dose can be translated to dietary therapies as well. For example, if a patient has responded to a milk elimination diet, we may offer them a liberalized milk free diet (23). This diet can include trace amounts of dairy and can avoid label reading for “may contain” or is low in the ingredient list. It allows a more sustainable food elimination balancing a reduced effectiveness. Additionally, liberalized food diet was often recommended in those at high risk of developing an IgE-mediated cow's milk allergy through strict avoidance, in an effort to prevent future conversion to anaphylactic responses (24, 25).

One must strike a careful balance between long term potential complications and histological remission with symptoms and current quality of life. Our approach focuses on engaging patients and families, explaining the chronic nature of the condition and finding common ground related to treatment and monitoring, using shared decision-making (19).

While overall remission rates of 39% are not noteworthy, there was a reduction in net eosinophils and an important outcome of decreased fibrostenotic disease represented by trachealization and stricturing was observed, which most would agree is the most feared complication of EoE (26).

The overall low remission rate cannot be fully explained by poor adherence or liberalized dosing and diet. This supports the necessity of further therapeutic options, such as biologics (27, 28).

Our study has several limitations including the lack of a fixed protocol (example medication dosage based on age). Another limitation of our data is the lack of consistent time frames, as some individuals have been followed for shorter durations, and we relied on the most recent endoscopy as a proxy for the best outcome for simplicity. Also, while counselling on EoE was done by the same group of providers, it was not scripted and individual factors such as background of food allergy, nutritional status or presence of a stricture would skew how treatment counselling was done.

A future direction is to collaborate with other major children's hospitals in Canada on a national EoE registry. In addition to a larger sample size, areas of improvement could include protocolization involving dose reduction, time windows for re-evaluation, qualitative studies to address barriers to adherence and further use of standardized disease severity scoring systems which include symptoms such as I-SEE (29).

## Conclusion

Despite certain shortcomings, our study provides unique information from a real world clinical setting (especially adherence data) and can serve in the establishment of quality benchmarks.

## Data Availability

The raw data supporting the conclusions of this article will be made available by the authors, without undue reservation.
